# Cloning and Overexpression of the *Toy* Cluster for Titer Improvement of Toyocamycin in *Streptomyces diastatochromogenes*

**DOI:** 10.3389/fmicb.2020.02074

**Published:** 2020-09-02

**Authors:** Zheng Ma, Yefeng Hu, Zhijun Liao, Jie Xu, Xianhao Xu, Andreas Bechthold, Xiaoping Yu

**Affiliations:** ^1^Zhejiang Provincial Key Laboratory of Biometrology and Inspection & Quarantine, College of Life Sciences, China Jiliang University, Hangzhou, China; ^2^Institute for Pharmaceutical Sciences, Pharmaceutical Biology and Biotechnology, University of Freiburg, Freiburg, Germany

**Keywords:** toyocamycin, *Streptomyces diastatochromogenes* 1628, cluster, heterologous expression, overproduction

## Abstract

The nucleoside antibiotic toyocamycin (TM) is a potential fungicide that can control plant diseases, and it has become an attractive target for research. *Streptomyces diastatochromogenes* 1628, a TM-producing strain, was isolated by our laboratory and was considered to be a potent industrial producer of TM. Recently, the putative TM biosynthetic gene cluster (*toy* cluster) in *S. diastatochromogenes* 1628 was found by genome sequencing. In this study, the role of *toy* cluster for TM biosynthesis in *S. diastatochromogenes* 1628 was investigated by heterologous expression, deletion, and complementation. The extract of the recombinant strain *S. albus*J1074-TC harboring a copy of *toy* cluster produced TM as shown by HPLC analysis. The Δcluster mutant completely lost its ability to produce TM. TM production in the complemented strain was restored to a level comparable to that of the wild-type strain. These results confirmed that the *toy* cluster is responsible for TM biosynthesis. Moreover, the introduction of an extra copy of the *toy* cluster into *S. diastatochromogenes* 1628 led to onefold increase in TM production (312.9 mg/l vs. 152.1 mg/l) as well as the transcription of all *toy* genes. The *toy* gene cluster was engineered in which the native promoter of *toyA* gene, *toyM* gene, *toyBD* operon, and *toyEI* operon was, respectively, replaced by *permE*^∗^ or SPL57. To further improve TM production, the engineered *toy* gene cluster was, respectively, introduced and overexpressed in *S. diastatochromogenes* 1628 to generate recombinant strains *S. diastatochromogenes* 1628-EC and 1628-SC. After 84 h, *S. diastatochromogenes* 1628-EC and 1628-SC produced 456.5 mg/l and 638.9 mg/l TM, respectively, which is an increase of 2- and 3.2-fold compared with the wild-type strain.

## Introduction

*Streptomyces* spp. are Gram-positive bacteria with a complex morphological differentiation and a highly developed secondary metabolism (Li and Tan, 2017; Jones and Elliot, 2018). They are well-known to produce a wide variety of bioactive natural products including antibiotics (Baltz, 2016; Kemung et al., 2018; Liu et al., 2018). Strain improvement is main goal of fermentation-based antibiotics production (Wang et al., 2010; Yamada et al., 2015). With the development of genetic engineering and metabolic engineering in *Streptomyces* spp. and an increasing knowledge of biosynthetic pathways, enhancement of antibiotic production has been addressed by using a rational approach (Chen et al., 2010; Tan et al., 2015; Palazzotto et al., 2019). Genetic manipulations of regulators, controlling the production of antibiotics or key structural genes governing the biosynthesis of antibiotics, are common and generally applicable strategies to achieve high-level production of antibiotics (Vicente et al., 2014; Martínez-Burgo et al., 2015; Zhu et al., 2017).

Because of its broad range of biological activities against pathogenic fungi, the nucleoside antibiotic toyocamycin (TM) has become an interesting target for research. TM is produced by *Streptomyces diastatochromogenes* 1628, but the production level is always very low (Ma et al., 2014a). In this context, efforts to improve TM production have been made by the overexpression of structural genes (Tao et al., 2016) or favorable genes (Ma et al., 2014a; Ma et al., 2014b) and the pathway-specific regulatory gene *toyA* (Xu et al., 2019) or ribosome engineering technology (Ma et al., 2016). Expression of an extra copy of *toyG* and *frr* led to a 65.5% increase in TM production (Tao et al., 2016). In another study, expression of an extra copy of pathway-specific regulator *toyA* driven by different promoters led to up to twofold increase in TM production (Xu et al., 2019).

Despite this, it cannot be ignored that more than a single rate-limiting step is often in the biosynthetic pathway of secondary metabolites that leads to the complexity of genetic manipulations. As almost all genes responsible for the biosynthesis of secondary metabolites are clustered together, the introduction of an extra copy of the entire biosynthetic gene cluster into the parent strain is considered to be an effective strategy to increase the yield of antibiotics, in which regulatory genes, structural genes and antibiotic-resistant genes are expressed simultaneously to maintain their compatibility. Successful experiments have confirmed this strategy (Liao et al., 2010; Murakami et al., 2011; Du et al., 2013; Jiang et al., 2013; Zhou et al., 2014; Li et al., 2017). For example, Liao et al. (2010) introduced an extra copy of the nikkomycin biosynthetic gene cluster and this led to a 4-fold and 1.8-fold increase in nikkomycin Z and nikkomycin X, respectively. In the work described by Zhou et al. (2014), three to five copies of validamycin (VAL-A) biosynthetic gene cluster were integrated into *S. hygroscopicus* 5008 and this led to a 34% enhancement of VAL-A production in shake-flask fermentation. In addition, the work of Li et al. (2017) led to an improved pristinamycin II production which was achieved by the introduction of five copies of the pristinamycin cluster.

**FIGURE 1:**
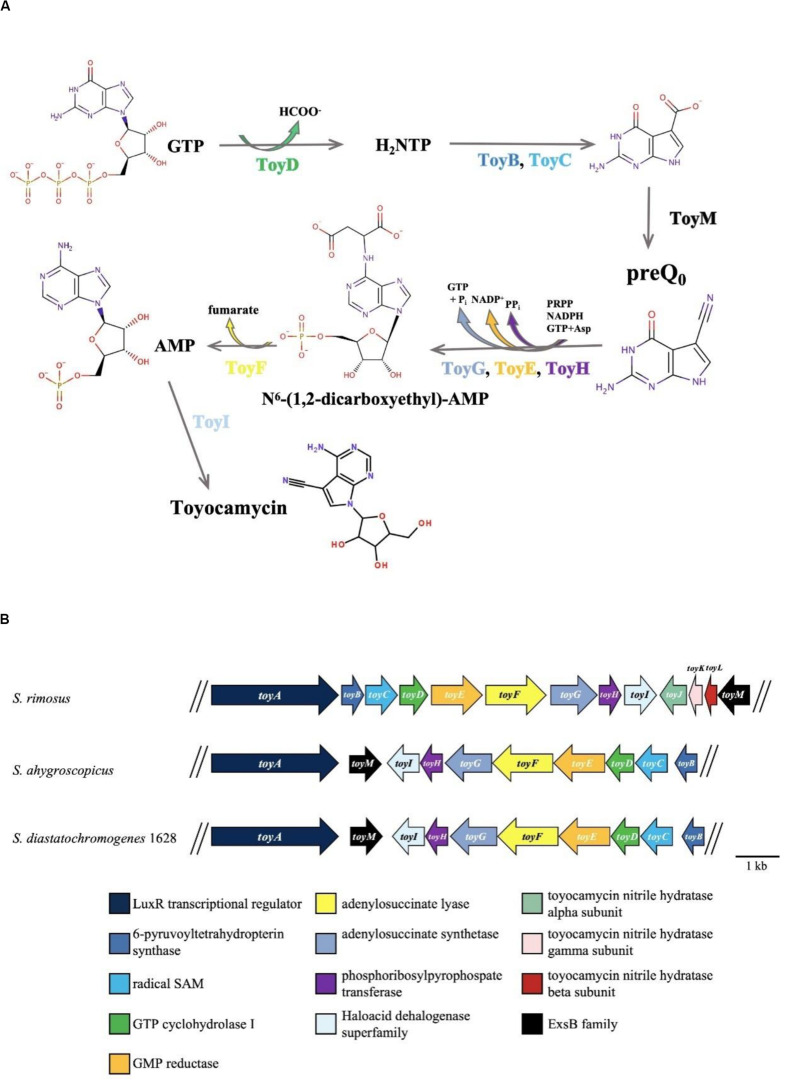
TM biosynthesis pathway and involved genes. **(A)** Putative TM biosynthesis pathway in *S. rimosus* (McCarty and Bandarian, 2008). **(B)** Organization of the toyocamycin gene clusters from *S. rimosus* (*upper*), *S. ahygroscopicus* (*middle*), and *S. diastatochromogenes* 1628 (*lower*).

Recently, the draft genome sequence of *S. diastatochromogenes* 1628 was obtained (unpublished). After analysis of the genome sequence by antiSMASH, preliminary data indicated the presence of 44 putative gene clusters including the TM biosynthetic gene cluster (*toy* cluster), which ranges from *toyA* to *toyI* (GenBank accession No. KY022432). Ten genes were controlled by four units: *toyA*, *toyM*, *toyEFGHI* operon (*toyEI*), and *toyBCD* operon (*toyBD*) (Figure 1). Surprisingly, each gene in the *toy* cluster showed the highest similarity to a corresponding gene from *S. ahygroscopicus* (JX576291) (Supplementary Table S1). However, thus far, the relationship of this published *toy* gene cluster with TM biosynthesis in *S. ahygroscopicus* has yet to be reported. Moreover, the overall gene organization and the sequence of the *toy* gene cluster differs from the gene organization and sequence of the *toy* gene cluster (GenBank accession No. EU573979) (Supplementary Table S1) of *Streptomyces rimosus* (McCarty and Bandarian, 2008).

In this context, two obvious questions are: whether putative *toy* cluster is responsible for TM biosynthesis in *S. diastatochromogenes* 1628? Whether the amplification of *toy* cluster could enhance the TM production in *S. diastatochromogenes* 1628?

To address the first question, in this study, the relationship between *toy* cluster and TM biosynthesis in *S. diastatochromogenes* 1628 was revealed by gene deletion and complementation experiment. The role of *toy* cluster was also confirmed by its heterologous expression in *S. albus* J1074.

To address the second question, the native *toy* cluster was cloned and overexpressed in parent strain *S. diastatochromogenes* 1628, in order to improve TM production. Furthermore, to overexpress the *toy* cluster, gain higher transcription levels of all *toy* genes, and further enhance TM production, the native *toy* cluster was engineered by replacing the native promoters of *toyA*, *toyM*, and *toyEI*, and of the *toyBD* operon by the strong constitutive promoter p*erm*E^∗^ and the synthetic promoter SPL57, respectively. The higher TM production was achieved by introduction of an extra copy of engineered cluster comparing to that by native cluster.

## Materials and Methods

### Materials

Q5 High-Fidelity Master Mix with GC-buffer was purchased from NEB. PCR reagents, restriction endonucleases, Miniprep, and Gel Extraction kits were purchased from TaKaRa Biotechnology Co. Ltd. Oligonucleotide primer synthesis and DNA sequencing of PCR products were performed by Shanghai Sunny Biotechnology Co. Ltd. China.

### Bacterial Strains, Plasmids, and Primers

The plasmids and strains used in this study are listed in Table 1 and Supplementary Table S2. TM producer *S. diastatochromogenes* 1628 has been deposited in the China General Microbiological Culture Collection Center (CGMCC No. 2060) (Ma et al., 2014a). *E. coli* JM109 was used as general host for gene cloning. The methylation-deficient strain *E. coli* ET12567/pUZ8002 was used as the donor for plasmid transfer to *Streptomyces*.

**TABLE 1:** Strains used in this study.

**Strains**	**Description**	**Source or reference**
*E. coli* JM109	General cloning host	Our lab
*E. coli* ET12567 (pUZ8002)	*Cm*^r^, *Km*^r^, donor strain for conjugation	Our lab
*S. albus* J1074	Host for heterologous expression of gene cluster	Makitrynskyy et al., 2020
*S. diastatochromogenes* 1628	Wild-type strain, toyocamycin producer	CGMCC 2060
*S. albus* J1074-TC	Strain *S. albus* J1074 integrated with native *toy* cluster	This work
1628-Δcluster	*Toy* gene cluster disruption mutant, derived from 1628 strain	This work
1628-Δcluster/pSET152	Mutant 1628-Δcluster with integrative plasmid pSET152	This work
1628-Δcluster/pSET152::*ncluster*	*Toy* cluster complemented strain, mutant 1628-Δcluster with integrative plasmid pSET152::*ncluster*	This work
1628-pSET152	Strain *S. diastatochromogenes* 1628 with integrative vector pSET152	This work
1628-TC	Strain *S. diastatochromogenes* 1628 with integrative vector pSET152::*ncluster*, harboring extra copy of native *toy* cluster	This work
1628-EC	Strain *S. diastatochromogenes* 1628 with integrative vector pSET152::*ecluster*, harboring extra copy of engineered *toy* cluster	This work
1628-SC	Strain *S. diastatochromogenes* 1628 with integrative vector pSET152::*scluster*, harboring extra copy of engineered *toy* cluster	This work

Plasmids pSET152, pGUS-SPL-57, and pGUS-ermE^∗^ were described previously (Siegl et al., 2013).

### Media and Growth Conditions

*Escherichia coli* strains were cultured using liquid or solid Luria-Bertani medium containing appropriate antibiotics at 37°C. Antibiotics were used in the following concentrations: apramycin (100 μg/ml), chloramphenicol (25 μg/ml), ampicillin (100 μg/ml), and kanamycin (50 μg/ml). *S. diastatochromogenes* 1628 was incubated at 28 °C and grown in solid mannitol soya flour (MS) medium (Zhao et al., 2019) for sporulation and conjugation. To generate spores, *S. diastatochromogenes* 1628 and its derivates were spread on MS medium and incubated for 4–5 days at 28°C. Collected spores were washed with water and preserved in water/glycerol (1:1, v/v) at −80°C.

### DNA Manipulations

General procedures for DNA manipulation were conducted according to the method described by Sambrook and Russel (2001). Intergeneric conjugation of *Streptomyces* and *E. coli* was performed as described by Kieser et al. (2000).

### Amplification and Sequencing of *Toy* Cluster

The *toy* cassette1 (harboring *toyA* gene with its 300-bp promoter regions, *toyM* gene, *toyI* gene and *toyH* gene) and *toy* cassette2 (harboring *toyB* gene with its 300-bp promoter regions, *toyC*, *toyD*, *toyE*, *toyF*, and *toyG* genes) were obtained through PCR amplification by using genome DNA of *S. diastatochromogenes* 1628 as the template. The *toy* cassette1 and cassette2 were amplified using primers Pcass1-F/R and Pcass2-F/R, respectively (Supplementary Table S3). High-fidelity PCR was performed using Q5 DNA polymerase (NEB) to obtain DNA fragments used for plasmid construction. PCR amplification was started at 98°C for 30 s, followed by 30 cycles of denaturation at 98°C for 20 s, annealing at 64°C for 20 s, and extension at 72°C for 6 min. After 30 cycles, a 5-min extension at 72°C was performed. Plasmid pSET152 was digested with *Xba*I and *Eco*RV and was fused to *toy* cassette1 and cassette2, using the Gibson assembly method to generate the recombinant plasmid pSET152::*ncluster*. Sequencing of the inserted 11410-bp native *toy* gene cluster confirmed that the gene did not contain any mutations.

### Construction of *Toy* Cluster Disruption (ΔCluster) Mutant and Its Complementation

Disruption of the *toy* cluster was performed by gene replacement *via* homologous recombination as described by Xu et al. (2019). For construction of *toy* cluster-disruption (Δcluster) mutant, a 3.0-kb fragment upstream of the *toyA* start codon was amplified by PCR using Pup-F/R (Supplementary Table S3) and a 3.0-kb fragment downstream of the *toyB* start codon was amplified by PCR using Pdown-F/R (Supplementary Table S3). The kanamycin-resistance gene (*neo*) was amplified from plasmid pET28a (Novagen) by PCR using primers Pkan-F/R (Supplementary Table S3). The three resulting DNA fragments were ligated into the *Xba*I and *Eco*RV sites of pKC1132 to yield pKC1132-Δ*cluster* by using Gibson DNA assembly methods as described by Gibson et al. (2008). Subsequently, introduction of the constructed pKC1132-Δ*cluster* into the wild-type strain *S. diastatochromogenes* 1628 was conducted by intergeneric conjugation. The Δcluster mutants were named *S. diastatochromogenes* 1628-Δcluster. Mutants were selected by both apramycin sensitivity (Apr^s^) and kanamycin-resistance (Kan^r^). The genotype of mutant *S. diastatochromogenes* 1628-Δcluster was confirmed by PCR.

For the complementation of the *toy* cluster in *S. diastatochromogenes* 1628-Δcluster, the plasmid pSET152::*ncluster* and the empty vector pSET152 as a control were introduced into mutant *S. diastatochromogenes* 1628-Δcluster by conjugation, resulting in the complemented strain 1628-Δcluster/pSET152::*ncluster* and the control strain 1628-Δcluster/pSET152, respectively.

### Heterologous Expression/Overexpression in *S. albus* J1074/*S. diastatochromogenes* 1628

Introduction of the constructed pSET152::*ncluster* into *S. albus* J1074 and *S. diastatochromogenes* 1628 was conducted by conjugation to yield recombinant strains *S. albus* J1074-TC and *S. diastatochromogenes* 1628-TC, respectively. Recombinant strains were confirmed using apramycin resistance and PCR.

### Engineered *Toy* Cluster by Insertion of Promoter *permE*^∗^ and SPL57

The construction of plasmid pSET152::*ecluster* was conducted by the following steps. First, three DNA fragments (717 bp of *toyM* gene, 4893 bp of *toyEI* cassette, and 1768 bp of *toyBD* cassette) were amplified from *S. diastatochromogenes* 1628 genomic DNA by using primers P1/P2, P3/P4, and P5/P6 (Supplementary Table S4), respectively. These fragments were, respectively, digested with *Spe*I/*Eco*RV and ligated into corresponding sites of pGUS-ermE^∗^ to generate plasmids pM, pEI, and pBD, respectively, in each of which the *gusA* gene in pGUS-ermE^∗^ was removed and replaced by the *toyM* gene, *toyEI* operon and *toyBD* operon. Second, three DNA fragments p*erm*E^∗^-*toyM*, p*erm*E^∗^-*toyEI*, and p*erm*E^∗^-*toyBD* were amplified from pM, pEI, and pBD by using primers P7/P8, P9/P10, and P11/P12 (Supplementary Table S4), respectively. The obtained three DNA fragments were fused into the plasmid p*erm*E^∗^-toyA linearized by *Eco*RV using the Gibson assembly method to generate the recombinant plasmid pSET152::*ecluster*, in which the *toyA* gene, *toyM* gene, *toyBD* operon and *toyEI* operon were all driven by promoter *perm*E^∗^. The inserted genes or cassettes were sequenced using sequencing primers. Sequencing results confirmed that no mutations were introduced.

A similar procedure described in the construction of plasmid pSET152::*ecluster* was performed to construct plasmid pSET152::*scluster*. The *gusA* gene in plasmid pGUS-SPL57 was replaced by *toyM* gene, *toyEI* operon, and *toyBD* operon to yield plasmids p57M, p57EI, and p57BD, respectively, in each of which the *toyM* gene, *toyEI* operon, and *toyBD* operon were placed under the control of synthetic promoter SPL57. Three DNA fragments SPL57-*toyM*, SPL57-*toyEI*, and SPL57-*toyBD* were then amplified from p57M, p57EI, and p57BD by using primers P13/P14, P15/P16, and P17/P18 (Supplementary Table S4), respectively. These were fused into the plasmid pSPL57-*toyA* linearized by *Eco*RV using the Gibson assembly method to generate the recombinant plasmid pSET152::*scluster*, in which *toyA* gene, *toyM* gene, *toyBD* operon, and *toyEI* operon all driven by promoterSPL57. The inserted genes or cassettes were sequenced using sequencing primers. Sequencing results confirmed that no mutations were introduced.

### Overexpression of Engineered *Toy Cluster* in *S. diastatochromogenes* 1628 Strain

The constructed plasmids pSET152::*ecluster* and pSET152::*scluster* were introduced into *E. coli* ET12567/pUZ8002 and then transferred into *S. diastatochromogenes* 1628 by conjugation to generate recombinant strains *S. diastatochromogenes* 1628-EC and *S. diastatochromogenes* 1628-SC, respectively. The recombinant strains were selected in the presence of apramycin at concentration of 50 μg/ml. The genotype of recombinant strains was verified by PCR using specific primers, binding to the apramycin resistance gene as part of the plasmid, which integrated into the chromosome.

### Analysis of Gene Transcriptional Levels by qRT-PCR

The extraction of RNA, design of primers, and analysis of transcriptional level of *toy* genes were performed as described by Wang et al. (2018). The qRT-PCR experiments were carried out in triplicate using RNA samples from three independent experiments.

### Production and Analysis of TM

Production and HPLC analysis of TM were conducted according to the method described by Ma et al. (2014b). Liquid chromatography-mass spectrometry (LC-MS) was performed on an Agilent 6120 Quadrupole MSD mass spectrometer (Agilent Technologies, United States) equipped with an Agilent 1290 Series Quaternary LC system and an Eclipse Plus C_18_ column (100 × 2.1 mm, 1.8 μm).

### Statistical Analysis

All experiments were conducted at least three times, and the results were expressed as mean ± standard deviations. Statistical analysis was performed with Student’s *t*-test. Samples with *p* values < 0.05 were considered statistically significant.

## Results

### *Toy* Cluster Is Responsible for TM Biosynthesis by Heterologous Expression and Deletion

**FIGURE 2:**
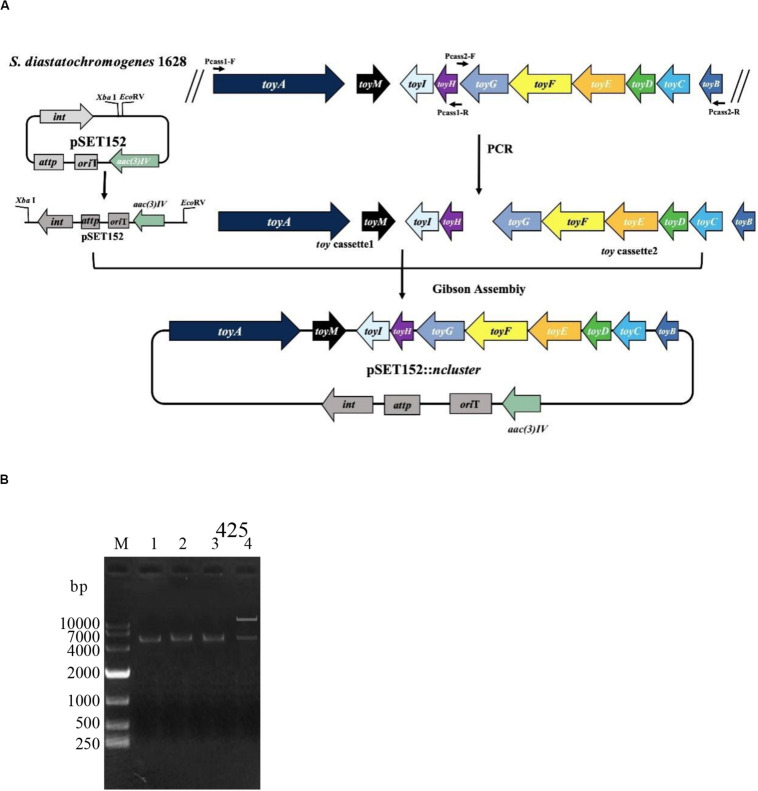
Schematic representation of the construction of pSET152::*ncluster*. **(A)** *Cassette1* harbors *toyA* gene with its 300-bp promoter regions, *toyM* gene, *toyI* gene and *toyH* gene; *Cassette2* harbors *toyB* gene with its 300-bp promoter regions, *toyC* gene, *toyD* gene, *toyE* gene, *toyF* gene, and *toyG* gene. Plasmid pSET152 was digested with *Xba*I and *Eco*RV and was fused to cassette1 and cassette2, using the Gibson assembly method to generate the recombinant plasmid pSET152::*ncluster*. **(B)** Identification of plasmid pSET152::*ncluster*. M: DL10000 DNA Marker. Lane 1, The PCR product of the 5.4-kb cassette1 were amplified by using the primers Pcass1-F/R; Lane 2, The PCR product of the 5.6-kb cassette2 was amplified by using the primers Pcass2-F/R; Lane 3, Plasmid pSET152 was digested and linearized by *Xba*I and *Eco*RV; Lane 4, The pSET152::*ncluster* was digested by *Xba*I and *Eco*RV.

**FIGURE 3:**
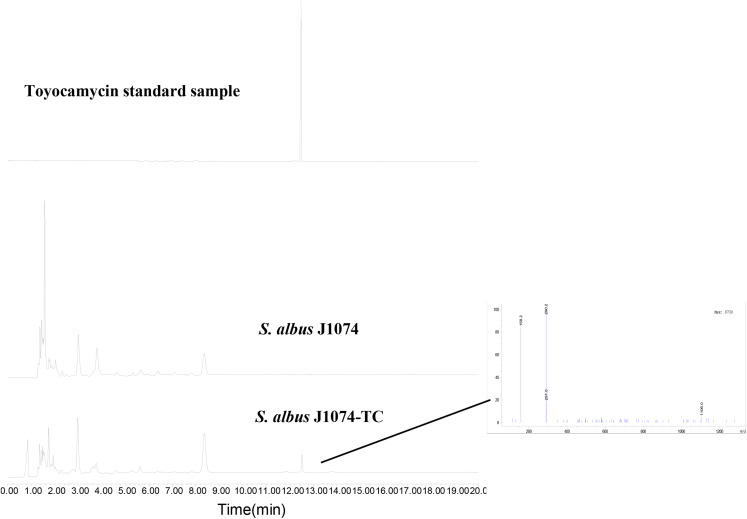
Heterologous expression of *toy* cluster in *S. albus* J1074. HPLC analysis of TM isolated from fermentation extracts of *S. albus* J1074 and recombinant strain *S. albus* J1074-TC; LC-MS (negative mode) of the distinct peak in the recombinant strain *S. albus* J1074-TC. LC-MS was performed on an Agilent 6120 Quadrupole MSD mass spectrometer (Agilent Technologies, United States) equipped with an Agilent 1290 Series Quaternary LC system and an Eclipse Plus C_18_ column (100 × 2.1 mm, 1.8 μm).

To investigate whether the putative *toy* cluster is involved in TM biosynthesis, a DNA fragment containing the putative *toy* cluster was cloned as described in the section “Materials and Methods.” To ensure the fidelity of the *toy* cluster sequence, amplification of the *toy* cluster was divided into cassette1 and cassette2, in which the original *toy* cluster sequences and direction from *toyA* to *toyB* of *S. diastatochromogenes* 1628 was retained. Then cassette1 and cassette2 were fused into plasmid pSET152 by Gibson assembly to generate recombinant pSET152::*ncluster* (Figure 2A). The plasmid pSET152::*ncluster* was confirmed by digesting with *Xba*I and *Eco*RV (Figure 2B) and was then passed through *E.coli* ET12567 (pUZ8002) and subsequently introduced into *S. albus*J1074 by intergeneric conjugation, to generate the recombinant strain *S. albus* J1074-TC resistant to 50 μg/ml apramycin. The integration of plasmid pSET152::*ncluster* into the chromosome of *S. albus* J1074 was verified by PCR assay (Supplementary Figure S1). In addition, the heterologous expression of *toy* gene cluster in *S. albus* J1074 was confirmed by quantitative RT-PCR analysis (Supplementary Figure S2). The recombinant strain *S. albus* J1074-TC and wild-type strain *S. albus* J1074 were cultured in shake-flask fermentation. As shown in Figure 3, culture filtrate from the recombinant strain *S. albus* J1074-TC was subjected to HPLC analysis, and a distinct peak appeared at a position corresponding to that of the standard sample of TM, whereas the culture filtrates from the negative control strains (*S. albus* J1074 and *S. albus* J1074-pSET152) lacked this peak. This peak was further identified as TM by means of LC-MS, whose negative molecular ion was at *m/z*290.2 [M-H]^–^ (Figure 3). Thus *S. albus* J1074-TC could produce TM, albeit in a small amount.

The *toy* cluster was completely replaced by a *neo* gene in the *S. diastatochromogenes* 1628 chromosome *via* double-crossover homologous recombination (Supplementary Figure S3A), to generate mutant *S. diastatochromogenes* 1628-Δcluster. The genotype of mutant 1628-Δcluster was verified *via* PCR analysis by using the primers PkanF/R, PkanF/PdownR, and PupF/PkanR, respectively. In mutant 1628-Δcluster, a single band 1.5-kb *neo* gene can be detected (Supplementary Figure S3B), suggesting that a double crossover event had occurred. Mutant 1628-Δcluster and wild-type strain *S. diastatochromogenes* 1628 were cultured in fermentation medium and their TM production was determined. HPLC analysis indicated that mutant *S. diastatochromogenes* 1628-Δcluster completely lost its ability to produce TM (Supplementary Figure S3C). The Δcluster mutant was then complemented by introducing pSET152::*ncluster*, to generate the complemented strain *S. diastatochromogenes* 1628-Δcluster/pSET152::*ncluster.* TM production in complemented strain *S. diastatochromogenes* 1628-Δcluster/pSET152::*ncluster* was restored to a level comparable to that in the wild-type strain *S. diastatochromogenes* 1628 under the normal fermentation condition (Supplementary Figure S3C). These results suggest that the cloned *toy* cluster is responsible for TM biosynthesis.

### Enhancement of TM Production by Addition of an Extra Copy of Native *Toy* Cluster in *S. diastatochromogenes* 1628

**FIGURE 4:**
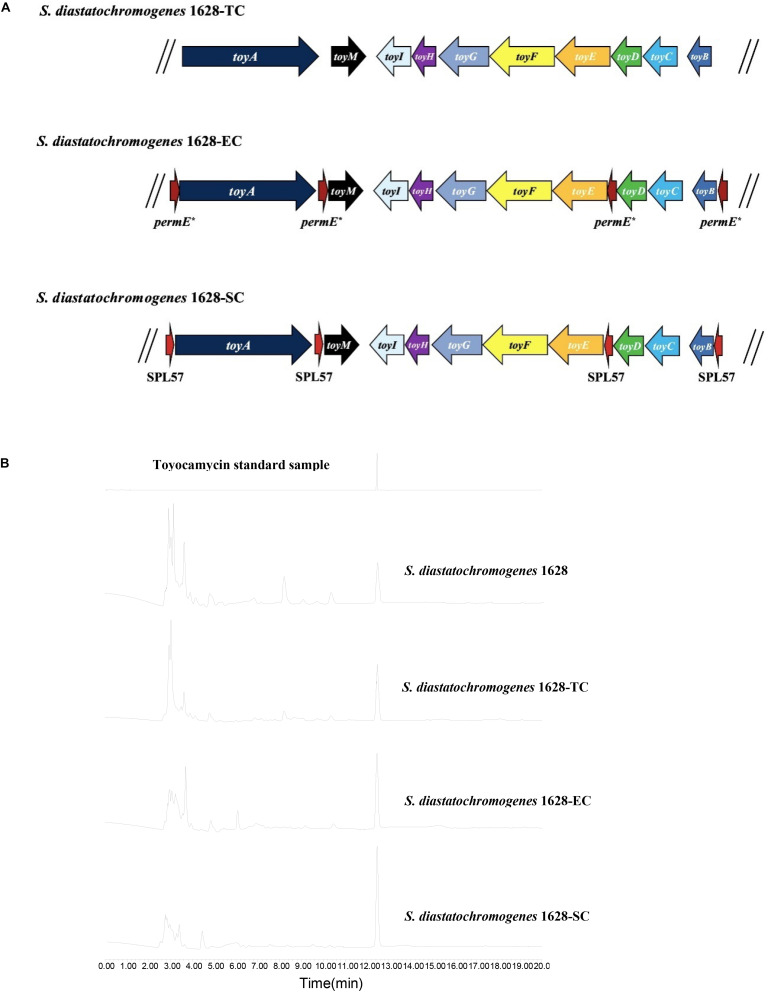
Characteristics of recombinant strains harboring an extra copy of *toy* cluster and their TM productions. **(A)** *S. diastatochromogenes* 1628-TC: *S. diastatochromogenes* 1628 harboring an extra copy of native *toy* gene cluster; *S. diastatochromogenes* 1628-EC: *S. diastatochromogenes* 1628 harboring an extra copy of engineered *toy* gene cluster in which four permE* promoters were inserted, the native promoter of *toyA* gene, *toyM* gene, *toyBD* operon and *toyEI* operon was, respectively, replaced by permE*; *S. diastatochromogenes* 1628-SC: *S. diastatochromogenes* 1628 harboring an extra copy of engineered *toy* gene cluster in which four SPL57 promoters were inserted, the native promoter of *toyA* gene, *toyM* gene, *toyBD* operon and *toyEI* operon was, respectively, replaced by SPL57. **(B)** HPLC analysis of TM isolated from fermentation extracts of the recombinant strain *S. diastatochromogenes* 1628-TC, *S. diastatochromogenes* 1628-EC, and *S. diastatochromogenes* 1628-SC.

We investigated whether an extra copy number of the native *toy* cluster increases TM production in *S. diastatochromogenes* 1628. The plasmid pSET152::*ncluster* was introduced into *S. diastatochromogenes* 1628 by intergeneric conjugation, to generate the recombinant strain 1628-TC (Figure 4A), which is resistant to 50 μg/ml apramycin. To verify the integration of plasmid pSET152::*ncluster* into the chromosome of *S. diastatochromogenes* 1628, genomic DNA of *S. diastatochromogenes* 1628-TC was isolated and used as template to perform PCR (Supplementary Figure S1). A large increase in TM production was observed for the recombinant strain 1628-TC harboring an extra copy of *toy* cluster (Figure 4B). After 84 h, the amount of TM produced by 1628-TC reached the highest level of 312.9 mg/l, a 105.7% increase in the yield of TM produced by *S. diastatochromogenes* 1628 (152.1 mg/l). In addition, introduction of either plasmid pSET152 or pSET152::*ncluster* had no effect on the cell growth of *S. diastatochromogenes* 1628 (Supplementary Figure S4).

**FIGURE 5:**
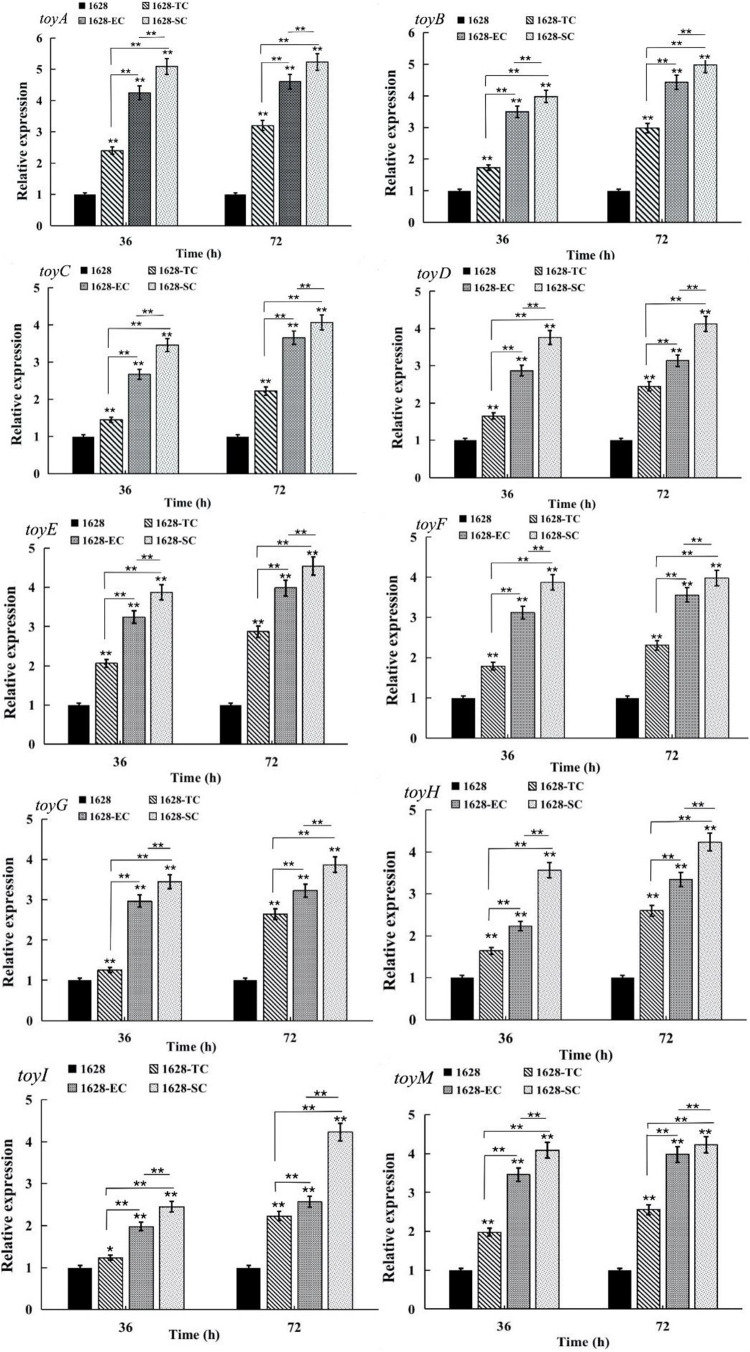
Comparison of the transcription levels of genes involved in TM production in different strains obtained by quantitative reverse transcription-PCR (qRT-PCR). 1628: *S. diastatochromogenes* 1628; 1628-TC: *S. diastatochromogenes* 1628-TC; 1628-EC: *S. diastatochromogenes* 1628-EC; 1628-SC: *S. diastatochromogenes* 1628-SC. The cells were harvested from the fermentation broth after 36 and 72 h. Error bars were calculated by measuring the standard deviations of the data from three replicates of each sample.(**) indicates highly statistically significant results (*P*-value < 0.01).

Quantitative real-time RT-PCR analysis was performed to assess the transcriptional levels of ten *toy* genes located in the *toy* cluster in recombinant strain 1628-TC and wild-type strain 1628, after 36, and 72 h of fermentation. Compared with wild-type, the transcriptional levels of all *toy* genes were greatly increased in the recombinant strain 1628-TC (Figure 5). We speculated that overexpression of native *toy* cluster thus enhances gene expression at the transcriptional level of all *toy* genes and further increases TM production in the recombinant strain 1628-TC.

### Engineered *Toy* Cluster and Its Overexpression to Improve TM Production

To overexpress the *toy* cluster, gain higher transcription levels of all *toy* genes, and further enhance TM production, we set out to engineer the native *toy* cluster as presented in Supplementary Figure S5. Briefly, *toyA* gene, *toyM* gene, *toyBD* operon and *toyEI* operon were placed under the control of constitutive promoter *permE*^∗^ or synthetic promoter SPL57. The introduction of the 4 *permE*^∗^ or SPL57 promoters and the assembly of four DNA fragments were conducted as described in the section “Materials and Methods,” yielding recombinant plasmids pSET152::*ecluster* and pSET152::*scluster*, respectively. Plasmids pSET152::*ecluster* and pSET152::*scluster* were introduced into *S. diastatochromogenes* 1628 by conjugation to generate recombinant strains 1628-EC and 1628-SC (Figure 4A), respectively.

Quantitative RT-PCR analysis revealed that the transcriptional levels of all the *toy* genes in *S. diastatochromogenes* 1628-EC and *S. diastatochromogenes* 1628-SC were markedly increased compared with those in *S. diastatochromogenes* 1628-TC, harboring an extra copy of native *toy* cluster (Figure 5). This result suggested that engineering *toy* cluster through insertion of promoter *permE*^∗^ or SPL57 successfully increased transcription of all the *toy* genes located in the *toy* cluster. We asked next whether the increased transcription of *toy* genes could improve the TM production.

**FIGURE 6:**
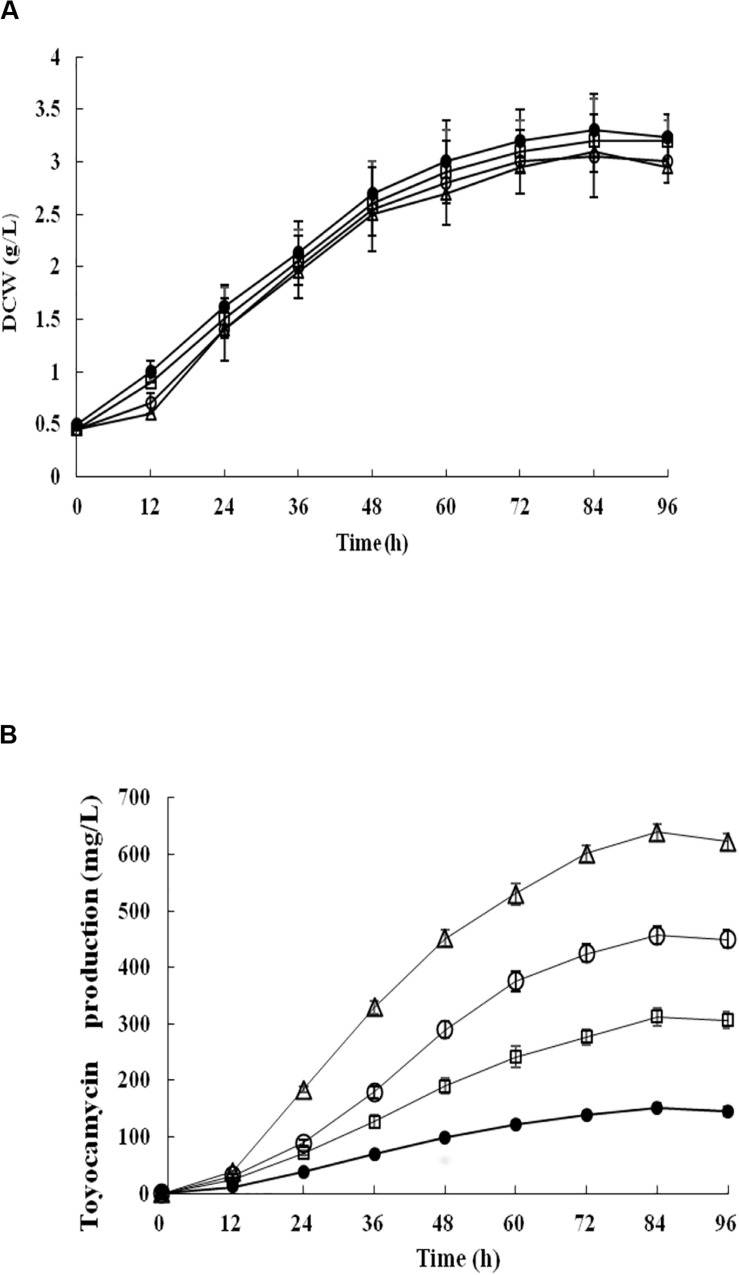
Detection and comparison of cell growth **(A)** and TM concentration **(B)** of the wild-type strain *S. diastatochromogenes* 1628 (filled circle), the recombinant strains *S. diastatochromogenes* 1628-TC (open square), *S. diastatochromogenes* 1628-EC (open circle) and *S. diastatochromogenes* 1628-SC (open triangle) in shake-flask culture experiment. All shake-flask fermentations were carried out in 250 ml flasks with a working volume of 40 ml at 200 rpm and 28°C. The medium was inoculated at 5% (v/v). Error bars were calculated from three different batches of fermentation.

Recombinant strains *S. diastatochromogenes* 1628-EC and *S. diastatochromogenes* 1628-SC, as well as *S. diastatochromogenes* 1628 as a control, were studied in shake-flask fermentation. As shown in Figure 6A, overexpression of engineered *toy* cluster had no significant effect on cell growth compared to that of the wild-type strain *S. diastatochromogenes* 1628. In contrast, it exhibited great promotional effect on TM production (Figure 4B). After 84 h, *S. diastatochromogenes* 1628-EC and 1628-SC increased TM levels to 456.5 mg/l and 638.9 mg/l, respectively (Figure 6B).

To determine the stability of engineered *toy* cluster in the chromosome of *S. diastatochromogenes* 1628, samples, taken from several continuous shake-flask fermentations of two recombinant strains *S. diastatochromogenes* 1628-EC and 1628-SC in the absence of apramycin, were streaked on the MS agar medium with apramycin (50 μg/ml), and the TM concentration was measured. The results showed that the cells still conferred apramycin resistance and exhibited a similar high level of TM production (data not shown). These results suggest that the integration of an extra copy of an engineered *toy* cluster in the chromosome of *S. diastatochromogenes* 1628 is genetically stable.

## Discussion

The nucleoside antibiotic TM is highly efficient against a broad range of plant pathogenic fungi. McCarty and Bandarian (2008) were the first to report the involvement of *toy* genes (*toy A* to *toyM*) in TM biosynthesis in *Streptomyces rimosus* (ATCC 14673). Putative functions of *toy* genes were analyzed. Four genes (*toyD*, *toyB*, *toyC*, and *toyM*) are responsible for the biosynthesis of 7-cyano-7-deazaguanine (preQ_0_). PreQ_0_ is converted to TM by ToyH, ToyE, ToyG, ToyF, and ToyI (McCarty and Bandarian, 2008; Mccarty et al., 2009; Battaglia et al., 2011). *S. diastatochromogenes* 1628 was shown to produce TM (Ma et al., 2014a). To support our research on TM biosynthesis, the genome of *S. diastatochromogenes* 1628 recently was sequenced, and the TM biosynthetic gene cluster (*toy* cluster) was predicted. Heterologous expression is an effective method to identify relationship between gene cluster and secondary metabolite. Therefore, to check whether the putative *toy* cluster was required for TM biosynthesis in *S. diastatochromogenes* 1628, we attempted to express the *toy* cluster in other *Streptomyces* spp. host cells as well.

Because of its fast growth and efficient genetic system, the *S. albus* J1074 strain is one of the most widely used for the heterologous production of bioactive natural products (Kallifidas et al., 2018; Myronovskyi et al., 2018). Therefore, in this study, *S. albus* J1074 was employed as a host for heterologous expression of the *toy* cluster. A DNA fragment harboring the entire *toy* cluster with the natural promoter was cloned and integrated into the chromosome of *S. albus* J1074 to generate *S. albus* J1074-TC. The strain was able to produce TM (16.9 mg/l) as shown by HPLC analysis. The lower production rate could be explained either by a lack of positive regulatory network or by an inadequate supply of important precursors. But our result clearly indicates that the *toy* cluster (GenBank accession No. KY022432) is involved in TM biosynthesis in *S. diastatochromogenes* 1628. It also suggests that differences in the location and orientation of genes in the known TM cluster do not affect their biological functions. Results were confirmed by gene deletion- and complementation-experiments.

The long-term goal of our research is to improve TM production of *S. diastatochromogenes* 1628 by rational design. This will eventually lead to an increased TM production and opens the way for more potent and useful TM analogs for industrial application.

Amplification of the entire biosynthetic gene cluster led to an improvement of the production of secondary metabolites in other strains (Du et al., 2015; Liu et al., 2017). Therefore, we constructed a strain (*S. diastatochromogenes* 1628-TC) harboring an extra copy of the *toy* cluster. TM production as well as the transcription of all *toy* genes was higher in this strain compared to that of wild-type strain *S. diastatochromogenes* 1628. To improve TM production further, we subsequently engineered the *toy* cluster to increase transcription of all *toy* genes. In our earlier study, the constitutive promoter *permE*^∗^, which is commonly used for gene expression in streptomycetes, was selected to increase the expression of favorable genes to improve TM production in *S. diastatochromogenes* 1628 (Ma et al., 2014b; Tao et al., 2016). Siegl et al. (2013) developed a synthetic promoter library for actinomycetes based on the −10 and −35 consensus sequences of the constitutive and widely used ermEp1 promoter. The synthetic promoters exhibited a wide range of promoter strengths (2% to 319%) compared with the ermEp1 promoter. Interestingly, promoter SPL-57 was shown to be much stronger than *permE*^∗^. Moreover, the synthetic promoter SPL57 showed high activity comparable with that of *permE*^∗^ in *S. diastatochromogenes* 1628 by using GUS as reporter gene (Xu et al., 2017). Recently, overexpression of the pathway-specific regulator *toyA* separately driven by the promoters *permE*^∗^ and SPL57 in *S. diastatochromogenes* 1628 led to a great increase in TM production compared to the 1628 strain (Xu et al., 2019). Replacement of native promoters with strong constitutive or synthetic promoters became a key strategy, resulting in an increase of gene transcription most probably by overcoming the regulatory barriers of the cell (Hammer et al., 2006; Myronovskyi and Luzhetskyy, 2016; Zhou et al., 2017; Horbal et al., 2018). In this study, the promoters *permE*^∗^ and SPL57 were introduced into the cluster resulting inpSET152::*ecluster* and pSET152::*scluster*. Notably, in pSET152::*ecluster* and pSET152::*scluster, toy* genes are arranged in tandem in the same direction as in the native *toy* cluster. Only the native promoters of the *toyA* gene, *toyM* gene, *toyBD* operon and *toyEI* operon were replaced by *permE*^∗^or SPL57. Recombinant strains *S. diastatochromogenes* 1628-EC and 1628-SC increased TM levels to 456.5 mg/l and 638.9 mg/l, respectively, resulting in a 2- and 3.2-fold increase compared to that of wild-type strain *S. diastatochromogenes* 1628, respectively. Meanwhile, the highest TM level produced by *S. diastatochromogenes* 1628-EC and 1628-SC was 45.9% and 104.2% higher than that produced by the *S. diastatochromogenes* 1628-TC (312.9 mg/l), respectively. In addition, we were able to show that *toy* genes were stronger expressed in these strains than in the wt strain. Based on our results we can conclude that the expression of clusters containing “artificial” promoters is more efficient than the expression of cluster containing the native promoters.

The largest improvement in TM production was detected in recombinant *S. diastatochromogenes* 1628-SC. This result shows that the promoter SPL57 is more suitable for the expression of the *toy* gene to improve TM production than the promoter *permE*^∗^. A similar phenomenon was also found in our previous study (Xu et al., 2019), in which overexpression of *toyA* driven by SPL57 exhibited a greater increase in TM production compared to that of *toyA* driven by *permE*^∗^. It is important to mention that co-expression of the whole cluster containing the “artificial” promoters led to a higher TM production than co-expression of only *toyA* behind an artificial promoter (using *permE*^∗^(456.5 mg/l vs. 309.3 mg/l; or SPL57 (638.9 mg/l vs. 456.3 mg/l). The stable integration of the engineered *toy* cluster in the chromosome of *S. diastatochromogenes* 1628 did not show any effect on cell growth.

In order to further optimize TM production, the engineered *toy* cluster was introduced into the resistant (Rif^r^) mutant *S. diastatochromogenes* 1628-T15 (Ma et al., 2016). Preliminary results indicate an explosive increase in TM production in this strain (data not shown). We are now planning to further investigate the strain, perform genome engineering (Huang et al., 2015; Li et al., 2017) and a precursor feeding strategy (Zabala et al., 2013; Gubbens et al., 2017; Zheng et al., 2019) to further improve TM production.

## Conclusion

This study confirmed that the putative TM biosynthetic gene cluster is responsible for TM biosynthesis in *S. diastatochromogenes* 1628. Amplification of specially engineered *toy* cluster greatly improved the TM production in *S. diastatochromogenes* 1628. This work importantly provides an effective strategy to improve TM production in *S. diastatochromogenes* 1628, and it can potentially be extended to other antibiotic overproduction strains.

## Data Availability Statement

The original contributions presented in the study are included in the article/Supplementary Material, further inquiries can be directed to the corresponding author.

## Author Contributions

ZM designed the research and wrote the manuscript. YH, ZL, JX, and XX conducted the experiments. AB revised the manuscript. XY checked the final version of the manuscript. All authors read and approved the manuscript.

## Conflict of Interest

The authors declare that the research was conducted in the absence of any commercial or financial relationships that could be construed as a potential conflict of interest.
