# Supplementary material for: Cloning and Overexpression of the Toy Cluster for Titer Improvement of Toyocamycin in Streptomyces diastatochromogenes
Source: Front Microbiol. 2020 Sep 2;11:2074. doi: 10.3389/fmicb.2020.02074 (PMC7492574; doi:10.3389/fmicb.2020.02074)
Supplement: Supplementary file 1 [file Data_Sheet_1.docx]

Supplementary Material

# Supplementary Figures and Tables

**
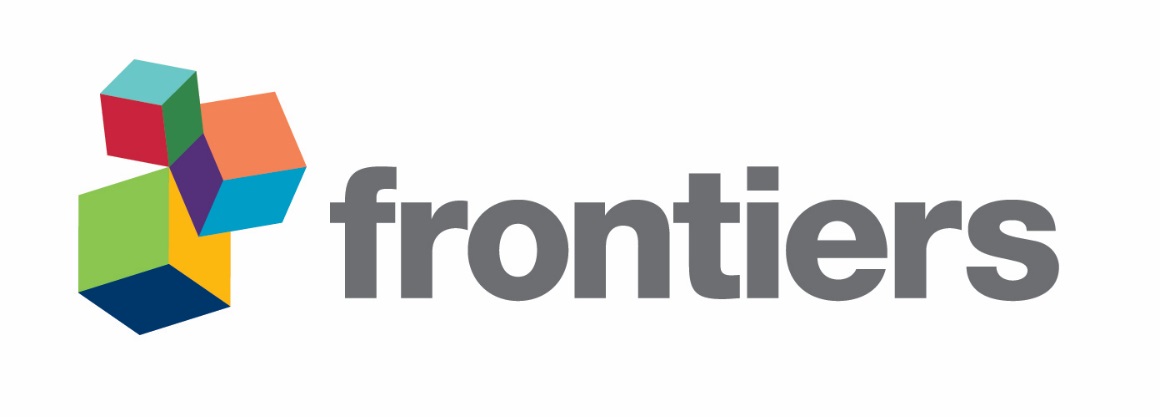
**

**Supplementary Figure 1.** PCR analysis of apramycin (*ap^r^*) gene from different recombinant strains. DL DNA 2000 marker was used (M). Lane 1: PCR product of *ap^r^* gene from *S. diastatochromogenes* 1628; lane 2: PCR product of *ap^r^* gene from plasmid pSET152; lane 3-4: PCR product of *ap^r^* gene from two randomly recombinant strains *S. albus* J1074-TC; lane 5-6: PCR product of *ap^r^* gene from two randomly recombinant strains *S. diastatochromogenes* 1628-TC; lane 7-8: PCR product of *ap^r^* gene from two randomly recombinant strains *S. diastatochromogenes* 1628-EC; lane 9-10: PCR product of *ap^r^* gene from two randomly recombinant strains *S. diastatochromogenes* 1628-SC.

**Supplementary Figure 2.** Comparison of the transcription levels of genes involved in TM production in different strains obtained by quantitative reverse transcription-PCR (qRT-PCR). 1628: *S. diastatochromogenes* 1628; J1074: *S. albus* J1074; J1074-TC: *S. albus* J1074-TC. The cells were harvested from the fermentation broth after 36 and 72 h. Error bars were calculated by measuring the standard deviations of the data from three replicates of each sample.(**) indicates highly statistically significant results (*P*-value < 0.01).

**
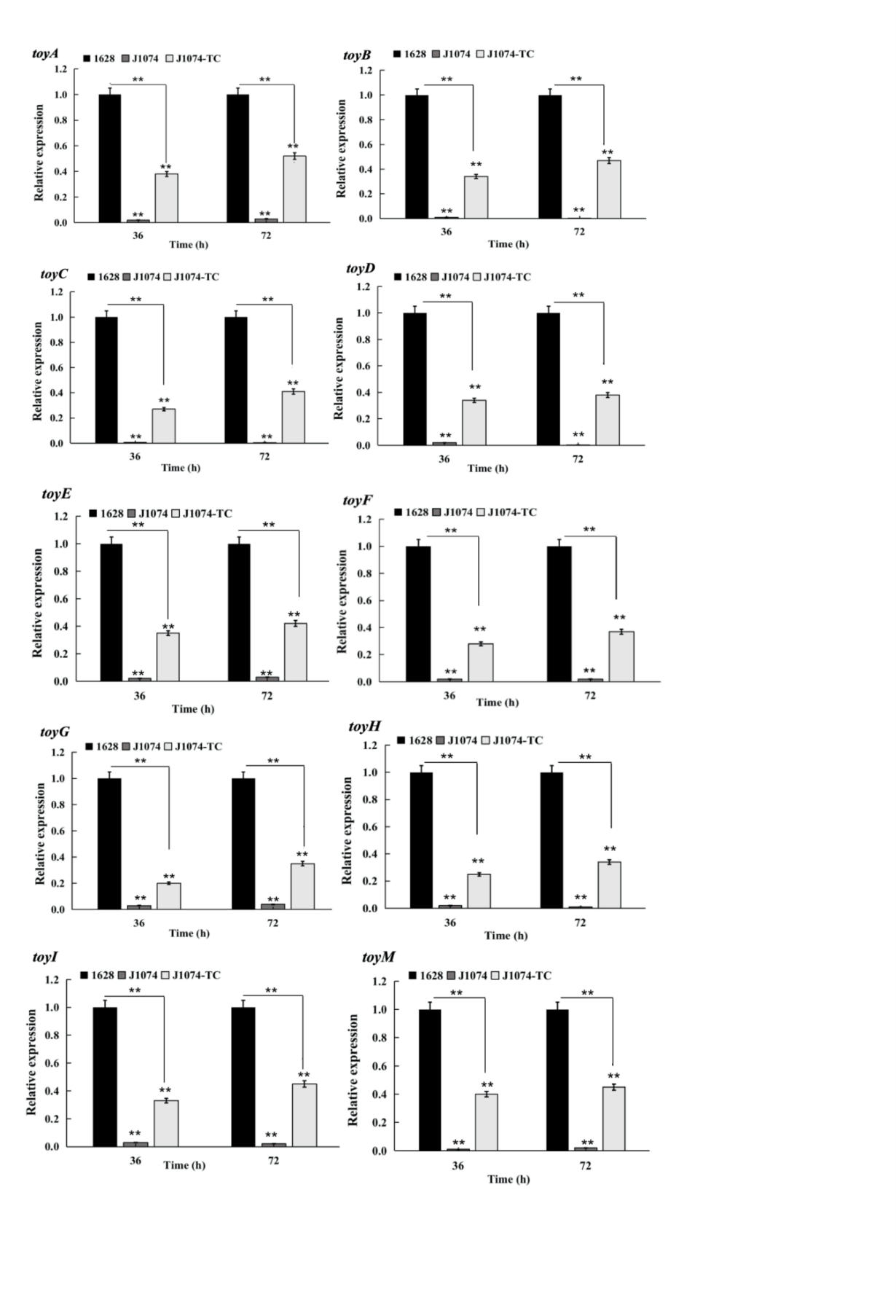
**

**Supplementary Figure 3.** Identification of function of *toy* cluster in *S. diastatochromogenes* 1628**. (A)** Schematic representation of the strategy used for deletion of the entire *toy* biosynthetic gene cluster. Disruption of *toy* cluster was performed by gene replacement *via* homologous recombination. **(B)** PCR verification of the mutant *S. diastatochromogenes* 1628-Δcluster. M: DL10000 DNA Marker. Lane 1, PCR product of the 16.4-kb using the primers Pup-F/Pdown-R from wt strain; Lane 2, PCR product of 6.0-kb using the primers Pup-F/Pdown-R from mutant 1628-Δcluster; Lane 3, PCR product of *neo* using the primers Pkan-F/R wt strain; Lane 4, PCR product of 1.5-kb *neo* gene using the primers Pkan-F/R from mutant 1628-Δcluster; Lane 5, PCR product of cassette containing 1.5-kb *neo* gene and 3.0-kb *toyA* upstream fragment using the primers Pup-F and Pkan-R from wt strain; Lane 6, PCR product of cassette containing 1.5-kb *neo* gene and 3.0- kb *toyA* upstream fragment using the primers Pup-F and Pkan-R from mutant 1628-Δcluster; Lane 7, PCR product of cassette containing 1.5-kb *neo* gene and 3.0-kb *toyB* downstream fragment using the primers Pkan-F and Pdown-R from wt strain; Lane 8, PCR product of cassette containing 1.5-kb *neo* gene and 3.0-kb *toyB* downstream fragment using the primers Pkan-F and Pdown-R from mutant 1628-Δcluster. **(C)** HPLC analysis of TM isolated from fermentation extracts of the wild-type strain *S. diastatochromogenes* 1628, *S. diastatochromogenes* 1628-Δcluster, *S. diastatochromogenes* 1628-Δcluster harboring pSET152, and *S. diastatochromogenes* 1628-Δcluster harboring pSET152::*ncluster.*

**A**

**
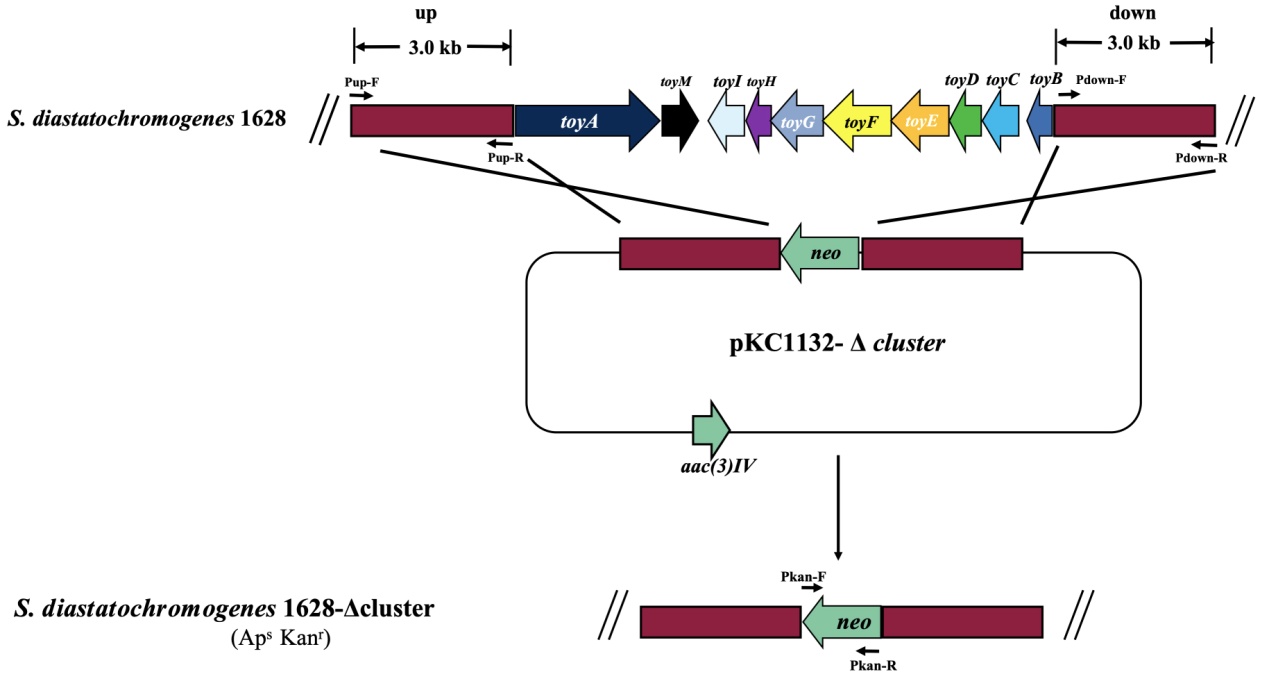
**

**B**

**C**

**Toyocamycin standard sample**

***S. diastatochromogenes* 1628**

***S. diastatochromogenes* 1628**

**-Δcluster**

***S. diastatochromogenes* 1628**

**-Δcluster/pSET152**

***S. diastatochromogenes* 1628**

**-Δcluster/pSET152::*ncluster***

**Supplementary Figure 4.** Detection and comparison of cell growth of wild-type strain 1628 (open square), 1628-pSET152 (filled square) and 1628-TC (open triangle) in shake-flask culture experiment. All shake-flask fermentations were carried out in 250 ml flasks with a working volume of 40 ml at 200 rpm and 28°C. The medium was inoculated at 5% (v/v). The error bars were calculated from three different batches of fermentation.

**Supplementary Figure 5.** Construction of the recombinant plasmid pSET152::*ecluster/scluster*. The procedure of construction of pSET152::*scluster* was as the same as pSET152::*ecluster*. In recombinant plasmid pSET152::*ecluster* or pSET152::*scluster* *toy* genes were arranged in tandem as the same direction as the native *toy* cluster.

**
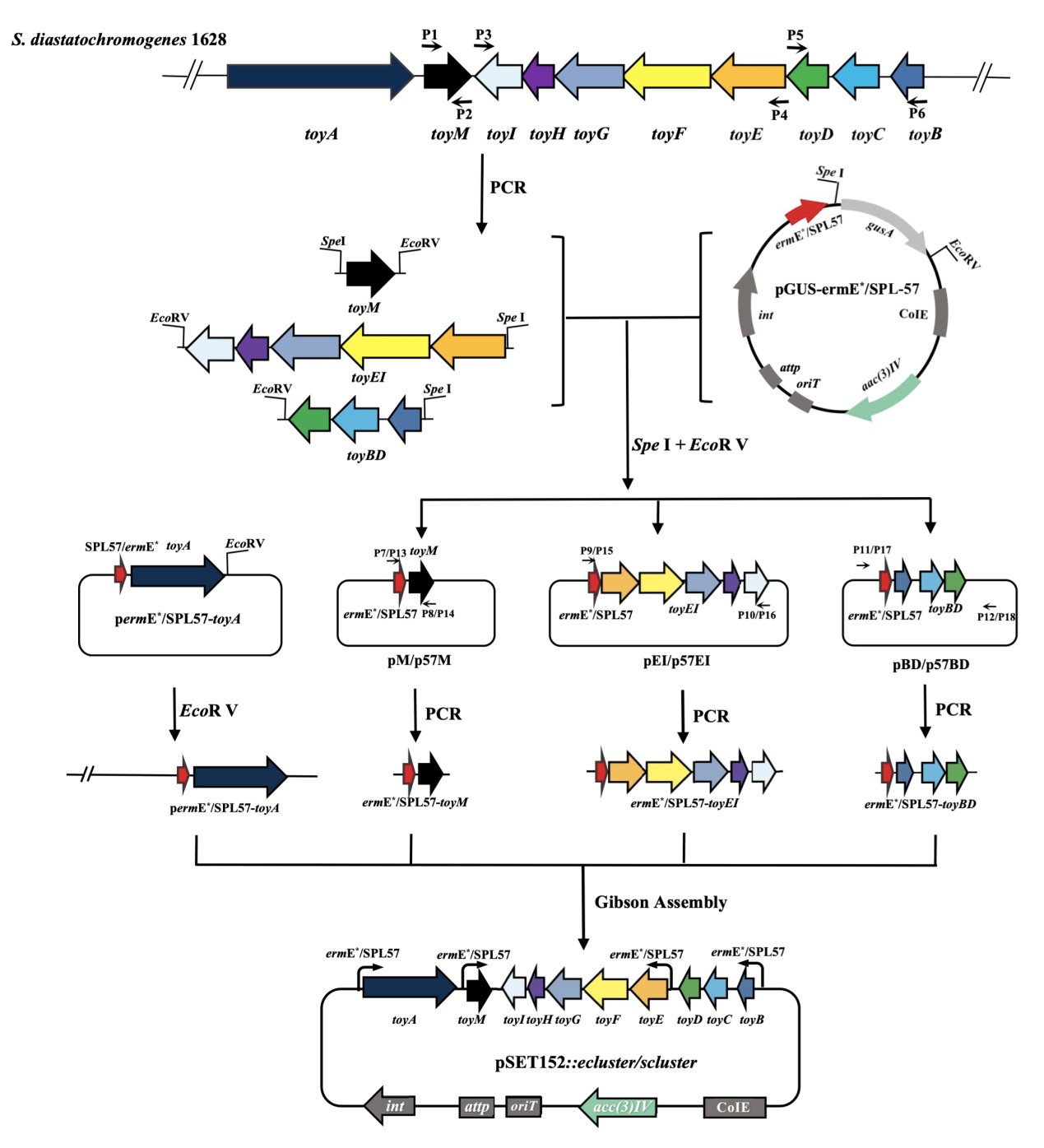
**

**Supplementary Table 1.** Comparison of corresponding toyocamycin biosynthetic genes from *S. rimosus*, *S. diastatochromogenes* 1628, and *S. ahygroscopicus*

| Gene | | Length  (bp) | Nucleotide  Identity | Amino acid  Identity | Putative function |
| --- | --- | --- | --- | --- | --- |
| *toyA_sd_*^a^  (2877 bp) | *toyA*^a^ | 2886 | 58.6 %  99.7 % | 33.9 %  99.9 % | LuxR transcriptional regulator |
|  | *tymA*^b^ | 2877 |  |  |  |
| *toyB_sd_*  (363 bp) | *toyB* | 396 | 71.9 %  100 % | 67.4 %  100 % | 6-pyruvoyltetrahydropterin synthase |
|  | *tymB* | 363 |  |  |  |
| *toyC_sd_*  (630 bp) | *toyC* | 621 | 79.0 %  99.7 % | 75.1 %  100 % | Radical SAM |
|  | *tymC* | 630 |  |  |  |
| *toyD_sd_*  (597 bp) | *toyD* | 603 | 75.2 %  98.2 % | 68.3 %  98.0 % | GTP cyclohydrolase I |
|  | *tymD* | 606 |  |  |  |
| *toyE_sd_*  (1146 bp) | *toyE* | 1155 | 76.5 %  99.8 % | 74.1 %  100 % | GMP reductase |
|  | *tymE* | 1146 |  |  |  |
| *toyF_sd_*  (1386 bp) | *toyF* | 1356 | 73.5 %  99.8 % | 41.9 %  100% | Adenylosuccinate lyase |
|  | *tymF* | 1386 |  |  |  |
| *toyG_sd_*  (1056 bp) | *toyG* | 1056 | 72.3 %  99.8 % | 68.8 %  100 % | Adenylosuccinate synthetase |
|  | *tymG* | 1056 |  |  |  |
| *toyH_sd_*  (522 bp) | *toyH* | 531 | 68.5 %  96.7 % | 59.3 %  97.1 % | phosphoribosyl-pyrophosphate transferase |
|  | *tymH* | 507 |  |  |  |
| *toyI_sd_*  (699 bp) | *toyI* | 726 | 67.6 %  94.3 % | 54.5 %  94.0 % | Haloacid dehalogenase superfamily |
|  | *tymI* | 660 |  |  |  |
| *toyM_sd_*  (717 bp) | *toyM* | 717 | 74.6 %  99.9 % | 72.4 %  100 % | ExsB family |
|  | *tymM* | 717 |  |  |  |

^a^ The sequence of *toy* genes involved in toyocamycin biosynthesis in *S. rimosus* has been cloned and submitted to the GenBank database under the accession number EU573979. The corresponding *toy* genes of S. *diastatochromogenes* 1628 were designated as *toyA_sd_* to *toyM_sd_* to distinguish the *toyA* to *toyM* genes of *S. rimosus*.

^b^The sequence of putative *tym* genes (*tymA*-*tymM*) involved in toyocamycin biosynthesis in *S. ahygroscopicus* has published in NCBI (GenBank accession No. JX576291)

**Supplementary Table 2.** Plasmids used in this study

| **Plasmids** | **Description** | **Source or reference** |
| --- | --- | --- |
| pSET152  pSET152::*ncluster*  pSET152::*ecluster*  pSET152::*scluster* | Integrative plasmid, Apr^r^, OriT RK2, ΦC31 *int/attP*  Plasmid pSET152 harboring *toy cluster* driven by its own promoter  Plasmid pSET152 harboring *toy cluster* in which *toyA, toyB, toyE and toyM* driven by promoter ermE^*^, respectively  Plasmid pSET152 harboring *toy cluster* in which *toyA, toyB, toyE and toyM* driven by synthetic promoter SPL57, respectively | Siegl et al. 2013  This work  This work  This work |
| pGUS-SPL57  pGUS-ermE^*^ | *gusA* under the control of synthetic promoter SPL57  *gusA* under the control of synthetic promoter p*erm*E^*^ | Siegl et al. 2013  Siegl et al. 2013 |
| pSPL57-*toyA* | Derived from pGUS-SPL57, *gusA* was replaced by *toyA* gene | Xu et al. 2019 |
| p*erm*E^*^-*toyA* | Derived from pGUS-ermE^*^, *gusA* was replaced by *toyA* gene | Xu et al. 2019 |
| pSPL57-*toyM* | Derived from pGUS-SPL57, *gusA* was replaced by *toyM* gene | This work |
| pSPL57-*toyBD* | Derived from pGUS-SPL57, *gusA* was replaced by *toyBCD* cassette | This work |
| pSPL57-*toyEI* | Derived from pGUS-SPL57, *gusA* was replaced by *toyEFGHI* cassette | This work |
| p*erm*E^*^-*toyM* | Derived from pGUS-ermE^*^, *gusA* was replaced by *toyM* gene | This work |
| p*erm*E^*^-*toyBD* | Derived from pGUS-ermE^*^, *gusA* was replaced by *toyBCD* cassette | This work |
| p*erm*E^*^-*toyEI* | Derived from pGUS-ermE^*^, *gusA* was replaced by *toyEFGHI* cassette | This work |
| pKC1132 | Conjugative plasmid, bearing Ap^r^, *rep*^pUC^ | Makitrynskyy et al. 2020 |
| pKC1132-Δ*cluster* | Derived from pKC1132, for deletion of *toy* cluster, containing up-stream homologous arms of *toyA* and down-stream homologous arms of *toyB,* and kanamycin-resistance gene (*neo*) | This work |

**Supplementary Table 3.**  Primers used for deletion of *toy* gene cluster in this study

| Primers | Sequence^a^ 5’-3’ |
| --- | --- |
| Pcass1-F | GGATGTGCTGCAAGGCGATTAAGTTGGGTAACGCCAGGGTTTTCCCAGTCACGACGTTGTAAAACGACGGCCAGTGCCAAGCTTGGGCTGCAGGTCGACTCTAGAccaaaggcgcgtaacggcaa |
| Pcass1-R | AGCGGCCAGGGAGGCGACCGatgagcggcacaccgatgtg |
| Pcass2-F | CCACATCGGTGTGCCGCtcatcggtcgcctccctggccgct |
| Pcass2-R | AGGCTTTACACTTTATGCTTCCGGCTCGTATGTTGTGTGGAATTGTGAGCGGATAACAATTTCACACAGGAAACAGCTATGACATGATTACGAATTCGATATCcgcctcccgagtgtcggg |
| Pup-F | GACGGCCAGTGCCAAGCTTGGGCTGCAGGTCGACTCTAGAcgtcctgggacttgtcgatg |
| Pup-R | CCTACACCGAACTGAGATACggagttcccctgtcacggct |
| Pdown-F | TTTCCCCGAAAAGTGCCACCgcagaccctcctgctgggata |
| Pdown-R | ACAGGAAACAGCTATGACATGATTACGAATTCGATATCggagcgcacggcgaacgtcc |
| Pkan-F | AGCCGTGACAGGGGAACTCCgtatctcagttcggtgtagg |
| Pkan-R | ATCCCAGCAGGAGGGTCTGCggtggcacttttcggggaaat |

^a^The underlined italic letters represent the restriction enzyme sites. The capital letters represent the universal assembly overlaps.

**Supplementary Table 4.** Primers used for construction of engineered *toy* cluster in this study

| Primers | Sequence^a^ 5’-3’ |
| --- | --- |
| P1 | *actagt*atggatcaagacgacagc(*Spe*I) |
| P2 | *gatatc*tcagggcgccgggtacgc(*Eco*RV) |
| P3 | *gatatc*cgccccgggcgcgcccggccccgccca(*Eco*RV) |
| P4 | *actagt*gtggcagttcgcgacatacgcaccg(*Spe* I) |
| P5 | *gatatc*gtgcgtactcctgtcgat(*Eco*RV) |
| P6 | *actagt*atggcttttcggatcacca(*Spe*I) |
| P7 | TGTGGCCGCTGCTGGTCGATTCCGCCGCCACCTGAGATtatgcatgcgagtgtccgttcg |
| P8 | GGCCGGGCGCGCCCGGGGCGtcagggcgccgggtacgccg |
| P9 | CGATCGACAGGAGTACGCACtatgcatgcgagtgtccgtt |
| P10 | CGGCGTACCCGGCGCCCTGAcgccccgggcgcgcccggcc |
| P11 | TCACACAGGAAACAGCTATGACATGATTACGAATTCGATtatgcatgcgagtgtccgttc |
| P12 | AACGGACACTCGCATGCATAgtgcgtactcctgtcgatcg |
| P13 | GCTGTGGCCGCTGCTGGTCGATTCCGCCGCCACCTGAGATttgaacggctggagggatac |
| P14 | GGCCGGGCGCGCCCGGGGCGtcagggcgccgggtacgccg |
| P15 | CGATCGACAGGAGTACGCACggggtgctgccgatcctggt |
| P16 | CGGCGTACCCGGCGCCCTGAcgccccgggcgcgcccggcc |
| P17 | TTCACACAGGAAACAGCTATGACATGATTACGAATTCGATggggtgctgccgatcctggt |
| P18 | ACCAGGATCGGCAGCACCCCgtgcgtactcctgtcgatcg |

^a^The underlined and italic letters represent the restriction enzyme sites. The capital letters represent the universal assembly overlaps.
